# Transcriptomic analysis reveals a previously unknown role for CD8^+^ T-cells in rVSV-EBOV mediated protection

**DOI:** 10.1038/s41598-017-01032-8

**Published:** 2017-04-20

**Authors:** Andrea R. Menicucci, Suhas Sureshchandra, Andrea Marzi, Heinz Feldmann, Ilhem Messaoudi

**Affiliations:** 1 Division of Biomedical Sciences, School of Medicine, University of California-Riverside, Riverside, CA 92521 USA; 2 Graduate Program in Genetics, Genomics and Bioinformatics, University of California-Riverside, Riverside, CA 92521 USA; 3 Laboratory of Virology, Division of Intramural Research, National Institute of Allergy and Infectious Diseases, National Institutes of Health, Hamilton, MT 59840 USA; 4 Department of Molecular Biology and Biochemistry, University of California-Irvine, Irvine, CA 92697 USA

## Abstract

Ebola virus (EBOV) poses a significant threat to human health as highlighted by the recent epidemic in West Africa. Data from animal studies and a ring vaccination clinical trial conducted in Guinea during the recent epidemic demonstrated that a recombinant VSV where G protein is replaced with EBOV GP (rVSV-EBOV) is safe and highly efficacious. We previously established that antibodies are essential for rVSV-EBOV mediated protection against EBOV; however, the mechanisms by which this vaccine induces a humoral response and the role of T-cells in rVSV-EBOV mediated protection remain poorly understood. Since this is the only vaccine platform that has completed Phase III clinical studies, it is imperative to gain a better understanding of its mechanisms of protection. Therefore, we performed a longitudinal gene expression analysis of samples collected from controls and T-cell-depleted macaques after rVSV-EBOV vaccination and EBOV challenge. We show that rVSV-EBOV vaccination induces gene expression changes consistent with anti-viral immunity and B-cell proliferation. We also report a previously unappreciated role for CD8^+^ T-cells in mediating rVSV-EBOV protection. Finally, limited viral transcription in surviving animals may boost protective responses after EBOV challenge by maintaining transcriptional changes. This study presents a novel approach in determining mechanisms of vaccine efficacy.

## Introduction

Ebola viruses are filamentous enveloped negative single-stranded RNA viruses with a 19 kb genome that can cause severe hemorrhagic fever (EHF) with case fatality rates reaching 90% depending on the species^1, 2^. EHF is characterized by an excessive inflammatory response, lymphocyte apoptosis, vascular impairment, and coagulation defects^3^. Ebola viruses continue to pose a significant threat to human health as evidenced by the recent epidemic in West Africa caused by a new strain of the species *Zaire ebolavirus* (EBOV), that lasted over 2 years, involved 10 countries, and resulted in ~28,600 total cases of EHF and ~11,300 deaths^4^.

This global health crisis led to the acceleration of several experimental therapeutics and vaccines for clinical trials. Two promising vaccine candidates have advanced the furthest in clinical trials. The recombinant chimapanzee adenovirus 3 vector (ChAd3) expressing EBOV GP (rChAd3-EBOV) is a replication-deficient vector that provides complete protection in cynomolgus macaques against ZEBOV challenge with a single dose^5^. This vaccine platform was reported to be well tolerated and immunogenic in a Phase 1/2a trial of 120 participants^6^. The second promising vaccine platform is the live-attenuated recombinant vesicular stomatitis virus (rVSV), which expresses EBOV GP in place of the VSV glycoprotein (rVSV-EBOV). A single dose of rVSV-EBOV provided 100% protection in nonhuman primates (NHPs) against EBOV challenge^7^. This vaccine provided protection lasting up to 12 months in mice and 18 months in guinea pigs^8^. Moreover, rVSV-EBOV provided complete and partial protection in cynomolgus macaques immunized 7 and 3 days before challenge, respectively^9^. Finally, a single dose of this vaccine conferred ~50% protection in rhesus macaques when administered up to 24 hours after EBOV challenge^10, 11^. As reported in multiple studies and clinical trials, this vaccine is safe, immunogenic and up to 100% efficacious in macaques and humans^12–15^.

We recently established antibody responses as the main mode of protection conferred by rVSV-EBOV^16^. In this study, groups of cynomolgus macaques were vaccinated with rVSV-EBOV and either depleted of CD4^+^ or CD8^+^ T-cells during vaccination or depleted of CD4^+^ T-cells during EBOV challenge. Only the animals depleted of CD4^+^ T-cells during vaccination and those that were vaccinated with rVSV expressing the *Marburgvirus* glycoprotein (rVSV-MARV; negative controls) succumbed to infection. Both of these groups lacked EBOV GP-specific antibodies, suggesting antibodies are required for rVSV-EBOV mediated protection^16^. However, the mechanisms by which this vaccine elicits a robust antibody response against EBOV GP and the contributions of T-cells to post challenge protection remain poorly understood.

To address these gaps, we carried out a longitudinal transcriptional analysis of peripheral blood mononuclear cells (PBMC) collected from these cynomolgus macaques after rVSV-EBOV immunization in addition to whole blood samples collected post EBOV challenge from control and depleted animals described in our earlier study^16^. Our analysis revealed temporary gene expression changes involved in innate immunity and cell cycle regulation, which may play a role in B-cell activation. Although both groups succumbed to challenge, CD4-depleted animals showed fewer differentially expressed genes (DEGs) 4 days post infection (dpi) and succumbed to infection 2 days later compared to negative control animals. Moreover, CD8-depleted animals showed greater DEGs than rVSV-EBOV vaccinated non-depleted (positive control) animals. Together these data indicate that CD8^+^ T-cells play a previously under-appreciated role in rVSV-EBOV mediated protection, albeit minimal. Lastly, protected animals exhibited lasting transcriptional changes along with the presence of intermittent low-levels of EBOV transcripts, suggesting that limited abortive viral transcription may enhance a protective host immune response following ebolavirus infection in vaccinated animals.

## Results

Blood samples were collected from our previous T-cell depletion study^16^ in which animals were vaccinated with: 1) rVSV-MARV (negative control); 2) rVSV-EBOV (positive control); 3) rVSV-EBOV and depleted of CD4^+^ T-cells (CD4-depleted); 4) rVSV-EBOV and depleted of CD8^+^ T-cells (CD8-depleted).

### Vaccination with rVSV-EBOV increases expression of genes involved in innate immune response and regulation of cell cycle

**Figure 1:**
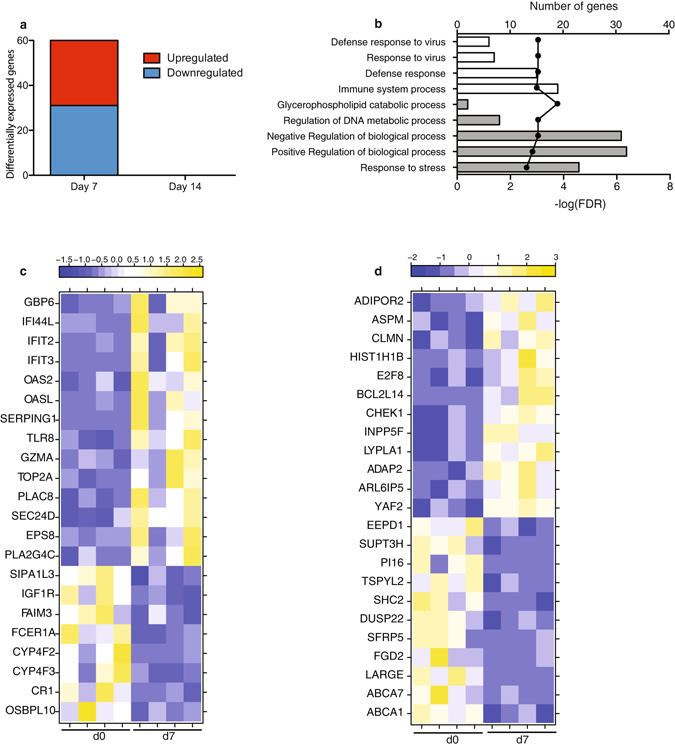
Vaccination with rVSV-EBOV induces expression of genes involved in innate immune response and cell cycle. (**a**) Number of differentially expressed genes (DEGs) defined as those with a fold change ≥ 2 and FDR corrected p-value ≤ 0.05; n = 4. (**b**) Bar graph depicting the most statistically significant Gene Ontology (GO) terms to which DEGs enriched; the line graph represents -log(FDR) of the enriched term. (**c**) Heatmap of all DEGs enriching to host defense GO terms (white bars) and biological process GO terms (grey bar); yellow represents increased expression while blue represents decreased expression; each column represents 1 animal. (**d**) Heatmap of all DEGs mapping exclusively to biological processes.

To determine the mechanisms by which rVSV-EBOV elicits protective humoral immune responses, we compared PBMC transcriptomes of non-depleted positive control animals on days 7 and 14 post vaccination relative to day of vaccination. At day 7 post-vaccination, 60 DEGs were detected (Fig. 1a), while no transcriptional changes were detected at day 14 (Fig. 1a). To view the distribution of DEGs induced by rVSV-EBOV across immune cell populations, we used the Immunological Genome Project Consortium (ImmGen) database^17^ (Supplementary Fig. 1). This analysis showed that vaccine-induced DEGs are mostly expressed in antigen presenting cells (dendritic cells, monocytes and macrophages), natural killer (NK) cells, and B-cells. To understand the biological impact of the gene expression changes, we performed functional enrichment using MetaCore^TM^. Of the 60 DEGs with human homologues, 46 enriched to several Gene Ontology (GO) processes that could be grouped into 2 themes: “host defense” and “biological processes” (Fig. 1b, white and grey bars respectively).

Upregulated DEGs that mapped to “host defense” GO terms (Fig. 1c) play a role in antiviral defense, such as interferon stimulated genes *IFI44L* (Interferon-induced Protein 44 Like; FC = 10.2), *OASL* (2′–5′-Oligoadenylate Synthetase-Like; FC = 3.7), and *IFIT2* (Interferon-induced protein with tetratricopeptide repeats 2; FC = 3.3) (Fig. 1c)^18^. Other upregulated DEGs play a role in innate immunity including *SERPING1* (Serpin family G member 1; FC = 145.7), *TLR8* (Toll-Like Receptor 8; FC = 2.4), and *GZMA* (Granzyme A; FC = 4.7); and DNA replication such as *TOP2A* (Topoisomerase (DNA) II Alpha 170 kDa; FC = 2.7). Downregulated genes that enriched to “host defense” regulate cell proliferation such as *FAIM3* (Fas Apoptotic Inhibitory Molecule 3; FC = 2.8), and inflammation e.g. *FCER1A* (Fc Fragment of IgE, High Affinity Ia; FC = 12.9).

All but one of the DEGs that enriched to “host defense” also mapped to those associated with “biological processes”. Several upregulated genes that mapped exclusively to “biological process” are also involved in regulation of cell cycle including *E2F8* (E2F Transcription Factor 8; FC = 6.7), *BCL2L14* (BCL2-Like 14; FC = 7.8), and *ASPM* (abnormal spindle microtubule assembly; FC = 3.6) (Fig. 1d). Other upregulated genes play a role in signaling including PI3K and AKT signaling molecule *INPP5F* (Inositol polyphosphate-5-phosphatase F; FC = 2.1)^19^ and GTPase activator *ADAP2* (ArfGAP with Dual PH Domains 2; FC = 3.2)^20^. Some of the downregulated genes are also involved in cell signaling such as *SHC2* (SHC adaptor protein 2; FC = 4.9) and *FGD2* (FYVE, RhoGEF and PH domain containing 2; FC = 6.3) (Fig. 1d).

### EBOV challenge results in larger transcriptional changes in animals that succumb to infection compared to those that survive

**Figure 2:**
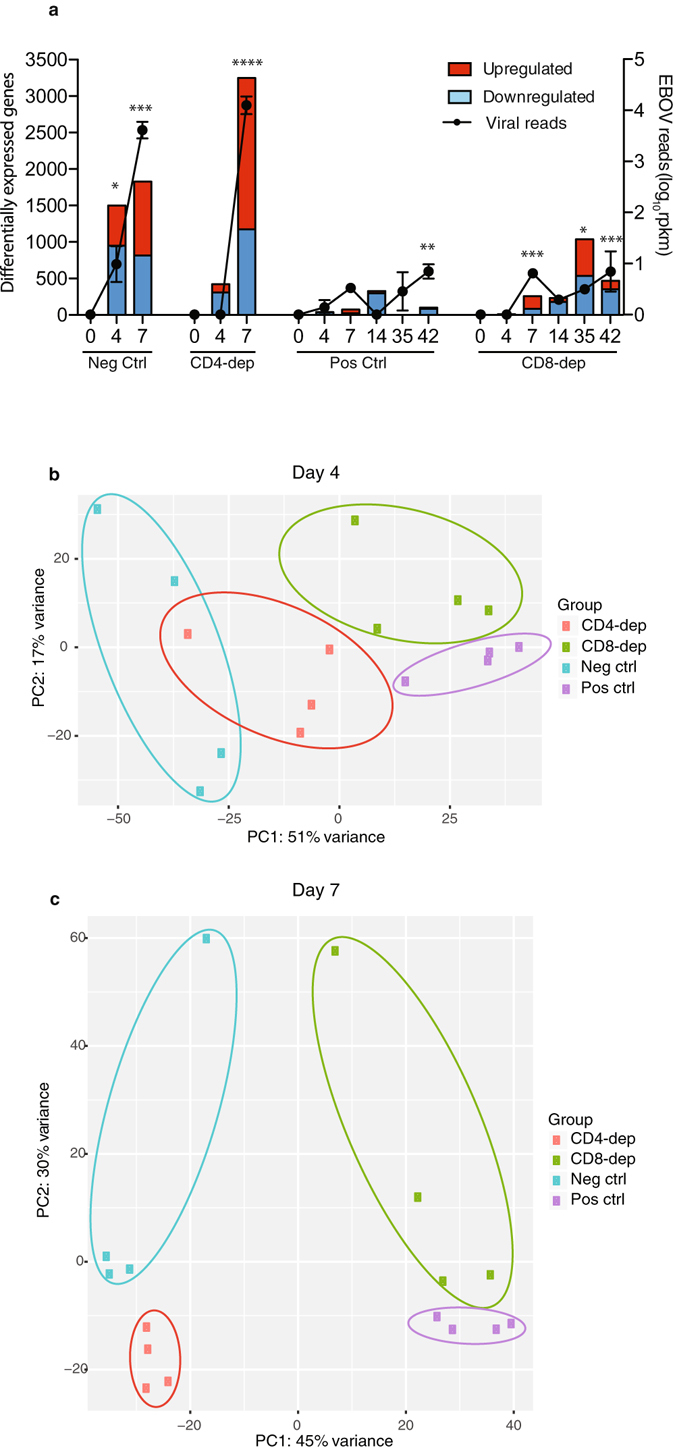
EBOV challenge results in larger transcriptional changes in animals that succumb to infection compared to those that survive. (**a**) Bar graph: Number of DEGs detected 4, 7 (or the day animals were necropsied), 14, 35 and 42 dpi in negative control, CD4-depleted, positive control, and CD8-depleted animals. Line graph: Total number of RPKM normalized transcripts mapping to the Kikwit-ZEBOV genome. Longitudinal changes of EBOV transcripts were carried out using one-way repeated measures ANOVA test followed by Dunnett’s multiple comparison post-test to determine differences between day 0 and subsequent days post-infection; *p < 0.05; **p < 0.01; ***p < 0.001; ****p < 0.0001; n = 4 in all groups. (**b**,**c**) Principal component analysis of all 4 groups 4 (B) and 7 (C) dpi.

To further investigate the correlates of rVSV-EBOV mediated protection, we characterized gene expression changes among the 4 groups of EBOV challenged animals (Fig. 2). The majority of the DEGs encoded protein with known human homologues (70–80%); some encoded non-coding RNA (1.5–8%); and the remaining were uncharacterized (10–25%) (Supplementary Table 1). For the remainder of the study we focused our analysis on protein coding DEGs with human homologs. In line with our previously published viral loads data^16^, only negative control animals showed a significant number of DEGs (1502) 4 dpi (Fig. 2a). Interestingly, although no viral replication or disease symptoms were detected in the CD4^+^ T-cell-depleted group 4 dpi, 422 DEGs were identified (Fig. 2a). Similarly, despite the lack of viremia and clinical scores, DEGs were detected in CD8-depleted (257) and positive control animals (74) 7 dpi, which persisted throughout the remainder of the study (Fig. 2a). RNA-Seq results were confirmed by measuring expression levels of *ARL6IP5*, *ATP5E*, *PLAC8*, *IFIT2* and *MX1* using qRT-PCR (Supplementary Fig. 2). Principal component analysis of transcriptional changes in all groups showed that negative control animals grouped closest to CD4-depleted animals (Fig. 2b), which was more pronounced on the days the animals were euthanized, in line with similarities in disease manifestation (Fig. 2c). Positive control and CD8-depleted animals clustered together 4 and 7 dpi, consistent with the lack of disease symptoms in these two groups (Fig. 2b,c).

We also determined total EBOV transcripts by mapping RNA-Seq reads to the EBOV genome (Fig. 2a) and each open reading frame (ORF) and intergenic region (IGR) (Supplementary Fig. 3). As expected, negative control animals had a high number of viral reads 4 dpi, which significantly increased 7 dpi (Fig. 2a and Supplementary Fig. 3). Despite the large number of DEGs 4 dpi, no EBOV transcripts were detected in CD4-depleted animals until 7 dpi when the number of EBOV transcripts in CD4-depleted animals was comparable to that measured in negative control animals. Interestingly, in positive control and CD8-depleted animals, very small numbers of transcripts were sporadically detected 7, 35 and 42 dpi (Fig. 2a and Supplementary Fig. 3).

### Negative control and CD4-depleted animals display different patterns of transcriptional changes 4 dpi, consistent with prolonged disease progression

**Figure 3:**
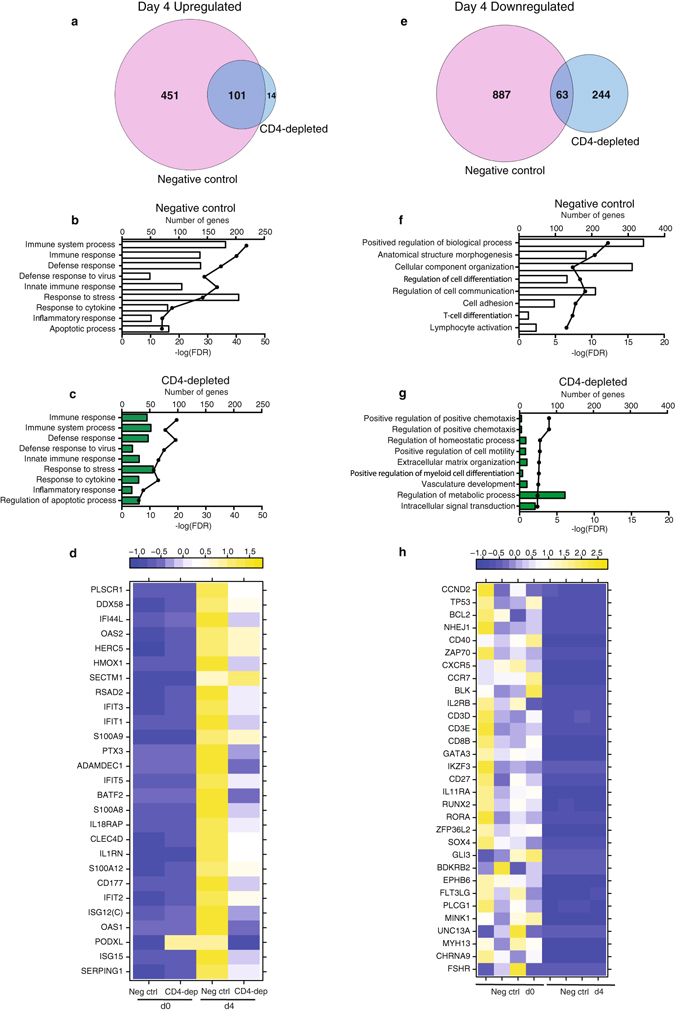
Partial protection in CD4-depleted group 4 dpi. (**a**) Venn diagram shows overlap between genes upregulated in negative control and CD4-depleted animals 4 dpi. (**b**,**c**) Bar graph depicting the most statistically significant GO terms to which upregulated DEGs in negative control animals (**b**) and CD4-depleted animals (**c**) enriched 4 dpi. (**d**) Heatmap of upregulated DEGs in both groups mapping to “Inflammatory Response” with a fold change cut off of 32 (based on negative control animals); each column shows median transcript RPKM counts of all 4 animals in each group. (**e**) Venn diagram comparing genes downregulated 4 dpi in negative control and CD4-depleted animals. (**f**,**g**) Bar graph depicting the most statistically significant GO terms to which down-regulated DEGs in negative control animals (**f**) and CD4-depleted animals (**g**) enriched 4 dpi. (**h**) Heatmap of down-regulated genes in negative control animals mapping to “Lymphocyte activation”, with a fold change cutoff of 15; each column represents 1 animal in the negative control group.

In line with a large overlap (Fig. 3a), DEGs upregulated 4 dpi in both nonsurviving groups showed similar functional enrichment (Fig. 3b,c). However, the negative control animals had significantly more DEGs mapping to these shared GO processes (Fig. 3b,c) with a higher fold change as illustrated by the heat map of shared DEGs mapping to “Inflammatory response” (Fig. 3d). In contrast, functional enrichment of down-regulated DEGs revealed distinct pathways in negative control and CD4-depleted groups, consistent with the limited overlap between the two sets of downregulated DEGs (Fig. 3e). Downregulated DEGs in negative control animals mapped to GO terms notably “Cell adhesion”, “T-cell differentiation” and “Lymphocyte activation” (Fig. 3f). Those DEGs include genes important for: T-cell activation e.g. *CD40* (FC = 348.51) and *ZAP70* (Zeta-Chain (TCR) Associated Protein Kinase 70 kDa; FC = 140.3); chemotaxis e.g. *CXCR5* (FC = 137.7) and *CCR7* (FC = 94.1); cell cycle e.g. *CCND2* (cyclin D2; FC = 55.7); apoptosis e.g. *BCL2* (B-Cell CLL/Lymphoma 2; FC = 37.4); and lymphocyte development such as *GATA3* (GATA Binding Protein 3; FC = 487.6) (Fig. 3h). On the other hand, downregulated genes in CD4-depleted animals enriched to cell migration, vasculature development and regulation of metabolic process including *F2RL1* (Coagulation Factor II Receptor-Like 1; FC = 122.39), *LAMB1* (Laminin, Beta 1; FC = 216.44), and *TCF7* (T-Cell-Specific Transcription Factor 1; FC = 24.58) (Fig. 3g).

### Negative control and CD4-depleted animals show transcriptional changes consistent with severe EBOV infection 7dpi

**Figure 4:**
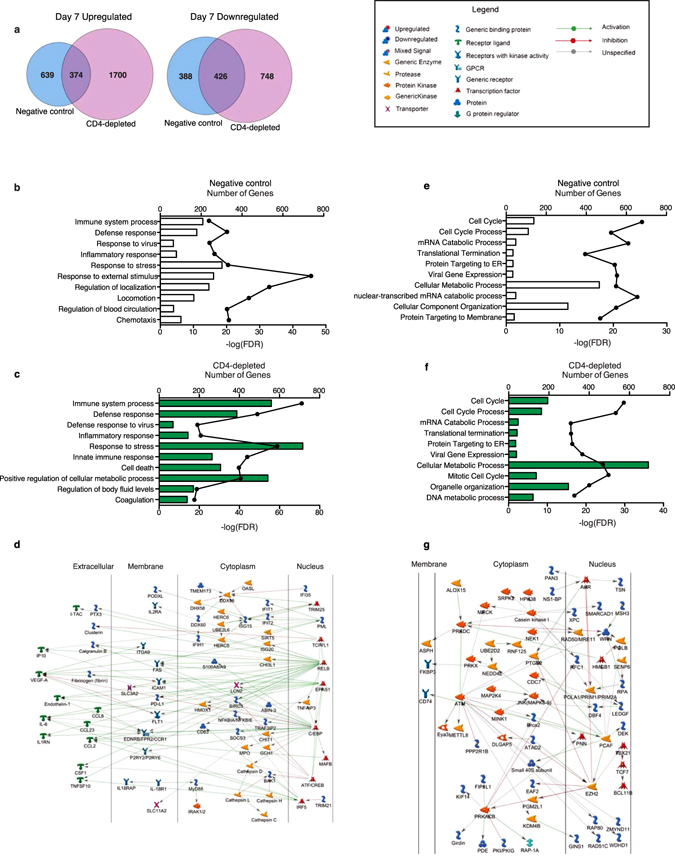
Negative control and CD4-depleted animals show transcriptional changes consistent with hemorrhagic fever on the day they succumb to EBOV challenge. (**a**) Venn diagram comparing upregulated and downregulated genes in negative control and CD4-depleted animals on the day of euthanasia. (**b**,**c**) Bar graph depicting the most statistically significant GO terms to which upregulated DEGs in negative control animals (**b**) and CD4-depleted animals (**c**) enriched on the day of euthanasia. (**d**) Network depicting direct interactions of common upregulated genes between negative control and CD4-depleted animals that map to “Immune System Process”. (**e**,**f**) Bar graph depicting the most statistically significant GO terms to which downregulated DEGs in negative control animals (**e**) and CD4-depleted animals (**f**) enriched on the day of euthanasia (**g**) Network depicting direct interactions of downregulated genes in both groups that map to “Cellular Metabolic Process”.

DEGs detected on the days both nonsurviving groups succumbed to infection showed significant overlap (Fig. 4a). Upregulated DEGs shared between the two groups enriched to GO terms associated with host defense and regulation of blood volume (Fig. 4b,c). Seventy-four genes upregulated in both groups and mapping to “Immune system process” showed direct interactions through two major transcription factors (Fig. 4d): *RELB* (V-Rel Avian Reticuloendotheliosis Viral Oncogene Homolog B) and *C/EBP* (CCAAT/Enhancer Binding Protein). Both *RELB* and *C/EBP* regulate chemokines and cytokines and their receptors e.g. *IP10*, *CCL8*, *IL-6* and *IL1RA* (Fig. 4d). Other upregulated DEGs in this network played a role in antiviral defense including *ISG15*, *IFIT1*, and *DDX58*.

Downregulated DEGs in both groups mapped to GO terms involved in cell division, metabolism, and translation (Fig. 4e,f). Sixty-eight genes downregulated in both groups and mapping to “Cellular metabolic process” showed direct interactions (Fig. 4g). Some of those genes were regulated by *EZH2* (Enhancer of Zeste 2 Polycomb Repressive Complex 2 Subunit) including Th1 cell-specific transcription factor *TBX21* (T-Box 21). Other genes in this network that were regulated by *PCAF* (Lysine acetyltransferase 2B) include ribosomal proteins that make up the Small 40 S subunit e.g. *RPS8* and *RPS25*. Other downregulated genes included regulators of signaling such as serine and threonine kinases *CSNK1G3* (Casein kinase I) and *PRKACB* (Protein Kinase, CAMP-Dependent, Catalytic, Beta).

### CD8^+^ T-cells play a role in rVSV-EBOV-mediated protection

**Figure 5:**
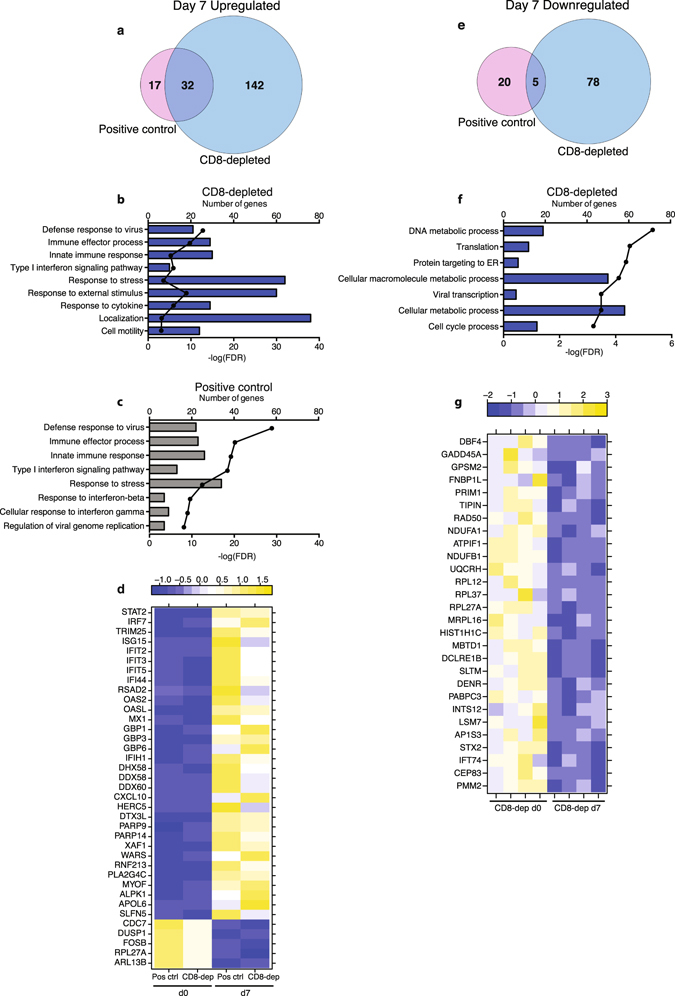
CD8^+^ T-cells play a role in rVSV-EBOV mediated protection. (**a**) Venn diagram of genes upregulated 7 dpi in positive control and CD8-depleted animals. (**b,c**) Bar graph depicting the most statistically significant GO terms enriched among up-regulated genes in CD8-depleted animals (**b**) and positive control animals (**c**) 7 dpi. (**d**) Heatmap of genes upregulated and downregulated in both CD8-depleted and positive control animals 7dpi; each column shows median transcript RPKM counts of all 4 animals in each group. (**e**) Venn diagram of genes downregulated in positive control and CD8-depleted animals 7 dpi. (**f**) Bar graph depicting the most statistically significant GO terms enriched among down-regulated genes in CD8-depleted animals 7 dpi. (**g**) Heatmap of all down-regulated genes, exclusive to CD8-depleted animals, mapping to all GO terms in (**f**) with a fold change cut off of 3; each column represents 1 animal in the CD8-depleted group.

A small number of DEGs detected 4 dpi in positive control animals, were mostly downregulated and involved in cell cycle processes (Supplementary Fig. 4a). On day 7, most of the upregulated genes in positive control animals were also upregulated in CD8-depleted animals (Fig. 5a), and similarly enriched to antiviral responses (Fig. 5b,c). The 32 common DEGs (Fig. 5d) include transcription factor *STAT2*, which regulates production of interferon-stimulated genes (ISGs). Indeed, several ISGs were upregulated including *ISG15*, *IFIT2* and *MX1* (MX Dynamin-Like GTPase 1). Moreover, genes encoding cytoplasmic sensors of viral nucleic acids e.g. *IFIH1* (Interferon-induced with Helicase C Domain 1) and *DHX58* (DEXH box helicase 58) were also upregulated (Fig. 5d).

At day 7, 25 genes that play a role in cell division were downregulated in positive control animals, notably transcription factors *HOXB8* (Homeobox B8; FC = 177.7), *EGR1* (Early growth response protein 1; FC = 18.7), and *FOSB* (FBJ Murine Osteosarcoma Viral Oncogene Homolog B; FC = 10.1) (Fig. 5e and Supplementary Fig. 4b). A higher number of downregulated DEGs (78) were detected in CD8-depleted animals that also enriched to DNA replication and cell cycle (Fig. 5f) including *PRIM1* (Primase, DNA, Polypeptide 1 (49 kDa); FC = 4.3) and *TIPIN* (TIMELESS Interacting Protein; FC = 3.9) (Fig. 5g). Other genes downregulated in CD8-depleted animals are involved in the electron transport chain such as *NDUFA1* (NADH:ubiquinone oxidoreductase subunit A1; FC = 4.3 and 6.3) and *ATPFIF1* (ATPase Inhibitory Factor 1; FC = 4.3) (Fig. 5g). Lastly, genes encoding ribosomal proteins and proteins involved in chromatin compaction such as *RPL12* (Ribosomal Protein L12; FC = 5.4) and *HIST1H1C* (Histone Cluster 1, H1c; FC = 3.9) were also downregulated (Fig. 5g).

### Gene expression changes persist in protected animals

**Figure 6:**
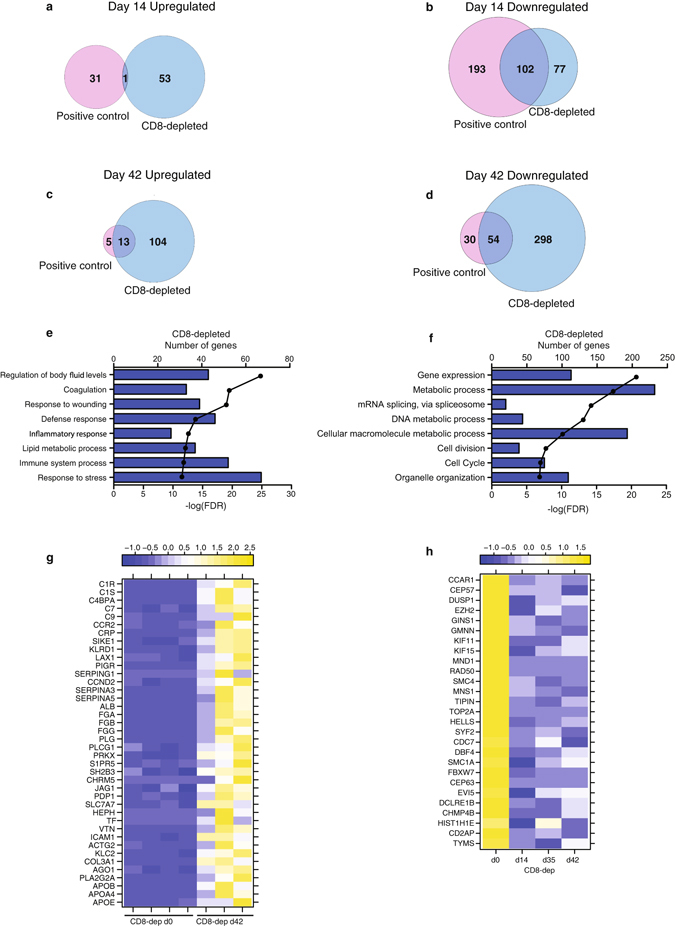
Gene expression changes persist in protected animals. (**a**) Venn diagram of genes upregulated 14 dpi in positive control and CD8-depleted animals. (**b**) Venn diagram of genes downregulated 14 dpi in positive control and CD8-depleted animals. (**c**) Venn diagram of upregulated genes 42 dpi in positive control and CD8-depleted animals. (**d**). Venn diagram of genes downregulated 42 dpi in positive control and CD8-depleted animals. (**e**,**f**) Bar graph depicting the most statistically significant GO terms enriched among upregulated genes (**e**) and downregulated genes (**f**) in CD8-depleted animals 42 dpi (**g**) Heatmap of genes upregulated 42 dpi in CD8-depleted animals mapping to “Coagulation” and “Immune System Process”; each column represents 1 animal in the CD8-depleted group (n = 3 at 42 DPI). (**h**) Heatmap of genes downregulated 42 dpi in CD8-depleted animals mapping to “Cell Cycle”; each column shows median transcript RPKM counts of all animals in the CD8-depleted group at each day.

DEGs upregulated 14 dpi in positive control and CD8-depleted animals enriched to different pathways, in line with the limited overlap between them (Fig. 6a and Supplementary Tables 2–3). Upregulated DEGs in positive control animals enriched to metabolic processes (Supplementary Table 2), while upregulated genes in CD8-depleted animals enriched to host defense (Supplementary Table 3). DEGs downregulated in both groups 14 dpi enriched to cell cycle processes (Fig. 6b and Supplementary Tables 2–3). DEGs were only detected in CD8-depleted animals 35 dpi (Supplementary Table 3, Supplementary Fig. 5a). DEGs upregulated in both positive control and CD8-depleted animals 42 dpi enriched to GO terms such as “Coagulation” (Fig. 6c,e; Supplementary Fig. 5b) and played a role in clotting notably, *FGA*, *FGB*, and *FGG* (Fibrinogen Alpha, beta, and gamma Chain) and *C9* (Complement component 9) (Fig. 6g and Supplementary Fig. 5c). As described 14 dpi, DEGs downregulated in both protected groups 42 dpi continued to map to cell cycle (Fig. 6d,f and Supplementary Fig. 6d). Indeed, several DEGs mapping to “Cell cycle” were downregulated 14 through 42 dpi in positive control (Supplementary Fig. 5e) and CD8-depleted animals (Fig. 6h) including *CCAR1* (Cell cycle and apoptosis regulatory protein 1), *CEP57* (Centrosomal protein 57 kDa), and *TOP2A* (Topoisomerase II alpha).

## Discussion

We previously established that antibody responses are essential for rVSV-EBOV mediated protection against EBOV infection, however, the mechanisms by which this vaccine induces a humoral response remain unclear. Furthermore, the role of T-cells in conferring protection by this vaccine is not completely understood. Therefore, we sought to better define the precise mechanisms underlying rVSV-EBOV mediated protection against EBOV challenge by conducting a longitudinal analysis of gene expression changes using RNA sequencing.

Vaccination with rVSV-EBOV induced acute gene expression changes that were only detectable 7 days post vaccination. These DEGs were associated with innate immune response, defense response to virus and regulation of cell proliferation. Specifically, we detected an upregulation of genes associated with ISGs important for antiviral defense (*OASL*, *IFIT2*, *IFI44L*, and *GBP6*). Previous studies have shown type I IFN signaling to be important in the generation of follicular helper T-cells, which promote B-cell differentiation into memory and plasma cells^21, 22^. Surprisingly, we did not detect genes involved in B-cell activation and antibody production. Using PBMCs may have precluded us from detecting a larger number of humoral immunity specific genes, since B-cells account for ~20% of PBMC. Thus, transcriptional changes exclusive to B cells may be diluted by the remaining immune cells in PBMC. Future studies will investigate changes in purified B-cells. Nevertheless, bioinformatics analysis using ImmGen showed that most of the cell cycle genes (*TOP2A*, *BCL2L14*, *ASPM*, and *CHEK1*) are predicted to be highly expressed by B cells. Increased expression of genes associated with cell proliferation preceded the T and B-cell proliferative bursts we previously reported 14 dpi^16^. We also detected increased expression of Granzyme A 7 dpi, suggestive of NK cell activation. These observations are in line with increased protein expression of type I IFN and cytokines associated with NK cell activation (IL-15 and IFNγ) that we recently reported in serum of cynomolgus macaques vaccinated within one week of EBOV challenge^9^.

Following EBOV challenge, we detected robust changes in gene expression in negative control animals that correlated with viremia and clinical scores. Surprisingly, we observed transcriptional changes in CD4-depleted animals 4 dpi despite no viremia and clinical symptoms. These data suggest that EBOV replicates in tissue reservoirs, such as draining lymph nodes. Upregulated DEGs 4 dpi in both nonsurviving groups mapped to similar GO terms. In line with disease severity, negative control animals had more DEGs that enriched to those GO terms and larger fold changes. In agreement with the different clinical status 4 dpi, downregulated genes enriched to different pathways in CD4-depleted and negative control animals. Specifically, expression of genes important for T-cell activation was significantly reduced in negative control animals, consistent with the earlier onset of clinical signs of EBOV infection including lymphopenia^16^. However, the delayed disease onset seen in the CD4-depleted animals is quickly lost as highlighted by similar transcriptomic profiles in negative control and CD4-depleted animals on the day of euthanasia. At this time point, DEGs in both groups mapped to immune system processes and inflammation, regulation of body fluids, cell cycle and metabolic processes, all of which are consistent with clinical features of EBOV infections.

DEGs involved in type I IFN signaling and host defense were detected in CD8-depleted and positive control animals 7 dpi despite the lack of viremia and disease signs, indicative of an antigen encounter. Indeed, we detected a sporadic small number of EBOV transcripts in samples from CD8-depleted and positive control animals 7 dpi. These observations are consistent with the detection of EBOV VP40-specific antibodies and increased EBOV-specific T-cell responses directed against antigens other than GP in positive control and CD8-depleted animals after EBOV challenge^16^. Therefore, limited abortive viral transcription may enhance the host immune response to EBOV infection. Interestingly, we detected a greater number of DEGs in CD8-depleted animals compared to positive control animals, especially at 7, 35 and 42 DPI. Similar to negative control and CD4-depleted animals, DEGs detected in CD8-depleted animals mapped to response to virus, cell cycle, translation and cellular metabolic processes albeit at much lower levels. Similarly, DEGs detected 42 dpi in positive control and CD8-depleted animals enriched to similar GO processes as DEGs detected in negative control and CD4-depleted at the time of euthanasia albeit at a much lower magnitude, including “cell cycle”, “metabolism”, “immune response”, and coagulation. Particularly interesting is that a majority of highly upregulated genes were involved in inflammation, cell adhesion, chemotaxis, and blood regulation.

In summary, our findings indicate that rVSV-EBOV vaccination induces robust innate responses that can modulate humoral response development. In addition, the detection of a higher number of DEGs in CD8-depleted animals (that enriched to similar GO processes as non-surviving animals) compared to positive control animals coupled with the delay in disease progression in CD4-depleted animals strongly suggest that CD8^+^ T-cell immunity engendered by rVSV-EBOV plays a previously under-appreciated role, albeit limited, in mediating protection against EBOV challenge. This is in contrast to rAd5 platform (rAd5-ZEBOV-GP) where CD8^+^ T cells play a critical role in mediating vaccine protection^23^. Finally, these data suggest that continuing host gene expression changes and low level abortive viral transcription may boost immune responses in protected animals.

## Materials and Methods

### Experimental Design

This study was designed to understand the mechanisms by which rVSV-EBOV provides protection against EBOV challenge. For this purpose, blood samples were collected from cynomolgus macaques derived from a previous T-cell depletion study^16^. RNA was extracted from PBMCs collected 0, 7 and 14 days post rVSV-EBOV vaccination (n = 4). RNA was also extracted from whole blood samples collected 0, 4, 7 days after EBOV challenge. The groups of EBOV challenged animals were as follows: 1) non-depleted, negative control animals vaccinated with rVSV-MARV (n = 4; all succumbed to challenge); 2) non-depleted, positive control animals vaccinated with rVSV-EBOV (n = 4; all survived challenge); 3) animals vaccinated with rVSV-EBOV and depleted of CD8^+^ T-cells during vaccination (n = 4; all survived challenge); 4) animals vaccinated with rVSV-EBOV and depleted of CD4^+^ T-cells during vaccination (n = 4; all succumbed to challenge). Additionally, RNA was extracted from whole blood samples collected 14, 35 and 42 days post EBOV challenge from all surviving animals (groups 2 and 3).

### Animal ethics statement

All Cynomolgus macaques from the previous study^16^ from which these samples were derived were handled in strict accordance with the recommendations described in the *Guide for the Care and Use of Laboratory Animals* of the National Institutes of Health, the Office of Animal Welfare, and the United States Department of Agriculture. Animal procedures were carried out under ketamine anesthesia by trained personnel under the supervision of veterinary staff, and all efforts were made to ameliorate the welfare and to minimize animal suffering in accordance with the “Weatherall report for the use of nonhuman primates” recommendations. All animal work was approved by the Institutional Animal Care and Use Committee at the Rocky Mountain Laboratories (RML). RML is accredited by the American Association for Accreditation of Laboratory Animal Care (Public Health Service Office of Laboratory Animal Welfare Animal Welfare Assurance numberA4149-01).

### Library generation and sequencing

RNA was isolated from PBMC and whole blood using the QIAmp Viral RNA Kit (Qiagen, Valencia, CA). RNA concentration and integrity was determined using an Agilent 2100 Bioanalyzer. Ribosomal RNA (rRNA) was depleted using the ClontechRibo-Gone rRNA Removal kit. Libraries were constructed using the ClontechSMARTer Stranded RNA-Seq kit. First, rRNA-depleted RNA was fragmented and converted to double stranded cDNA. Adapters were ligated and the ~300 base pair (bp) long fragments were then amplified by PCR and selected by size exclusion. Each library was prepared with a unique indexed primer for multiplexing. In order to ensure proper sizing, quantitation, and quality prior to sequencing, libraries were analyzed on the Agilent 2100 Bioanalyzer. Multiplexed libraries were subjected to single-end 100 bp sequencing using the Illumina HiSeq2500 platform.

### Bioinformatic analysis

Data analysis was performed with the RNA-seq workflow module of the systemPipeR package available on Bioconductor^24^. RNA-Seq reads were demultiplexed, quality filtered and trimmed. Three base pairs from the 5′ end were trimmed as per Clontech’s instruction and 30 bp from the 3′ end were trimmed based on quality for a total length of 67 bp per read. Quality reports were generated with the seeFastq function. Because genome annotation for *Macaca fascicularis* is not available, the *Macaca mulatta* genome sequence and annotation from Ensembl (Macaca_mulatta.MMUL_1.dna.toplevel.fa and Macaca_mulatta.MMUL_1.78.gtf), which has high sequence homology to *Macaca fascicularis*, was used. In order to determine the level of viral transcription at different time points, the EBOV strain Kikwit genome (Ebola virus/H.sapiens-tc/COD/1995/Kikwit-807223) from Virus Pathogen Resource was adjoined to the *Macaca mulatta* reference. ZEBOV open reading frames (ORFs), intergenic regions (IGRs) and leader and trailing sequences were defined based on the ZEBOV-Kikwit genome annotation GTF file: NP (469–2688), VP35 (3128–4150), VP40 (4478–5458), GP (6038–8067), VP30 (8508–9374), VP24(10344–11099), L (11580–18218), Leader (1–468), IGR_NP_VP35 (2689–3127), IGR_VP35_VP40 (4151–4477), IGR_VP40_GP (5459–6037), IGR_GP_VP30 (8068–8507), IGR_VP30_VP24 (9375–10343), IGR_VP24_L (11100–11579), Trailing (18219–18956). RNA-Seq reads were mapped with the alignment suite Bowtie2/Tophat2 against a reference genome containing both Macaca fascicularis and EBOV genome sequences. Raw expression values in the form of gene-level read counts were generated with the *summarizeOverlaps* function, counting only the reads overlapping exonic regions of genes, and discarding reads mapping to ambiguous regions of exons from overlapping genes. Normalization and statistical analysis of differentially expressed genes (DEGs) derived from either the animal or virus was performed using the *edgeR* package. Host DEGs were defined as those with a fold change ≥2 and a false discovery rate (FDR) corrected p value ≤ 0.05. Only protein coding genes with human homologs (Supplementary Table 1) were included for further analysis. Reads mapping to the EBOV genome were normalized by reads per kilobase of transcript per million mapped reads (RPKM). RNA-sequencing data presented in this article were submitted to the National Center for Biotechnology Information Sequence Read Archive (Accession number SRP090379). Venn diagrams, PCAs, and heatmaps were generated using R packages “VennDiagram”, “DESeq2” and “gplots”.

### Functional enrichment

Functional enrichment was done to identify clusters of genes mapping to specific biological pathways, specifically gene ontology (GO) terms, using MetaCore^TM^. Since this software requires human gene identifiers for analysis, rhesus DEGs were mapped to human homologs using BioMart (Supplementary Table 1).

### Gene expression confirmation by qRT-PCR

cDNA was synthesized using a High-Capacity cDNA Reverse Transcription Kit (Applied Biosystems, Foster City, CA) and expression levels were determined using TaqMan gene expression assays (Applied Biosystems). Fold changes were determined using the 2^(−ΔΔCT)^ ^25^ after normalization to expression levels of housekeeping gene ribosomal protein L32 (RPL32)^26^.

### Statistical analysis

Total normalized reads mapping to the EBOV genome were log_10_(x + 1) transformed. Longitudinal changes of the EBOV transcripts were carried out using one-way repeated measures ANOVA test followed by Dunnett’s multiple comparison post-test to determine differences between day 0 and subsequent days post-infection. Changes in gene expression via qRT-PCR were determined using a paired two-sided t-test. Statistical significance for all comparisons was determined at the alpha level of 0.05.

## Electronic supplementary material

Supplementary Information
