# Supplementary material for: Transcriptomic analysis reveals a previously unknown role for CD8+ T-cells in rVSV-EBOV mediated protection
Source: Sci Rep. 2017 Apr 20;7:919. doi: 10.1038/s41598-017-01032-8 (PMC5430516; doi:10.1038/s41598-017-01032-8)
Supplement: Supplementary file 1 — Supplementary Information [file 41598_2017_1032_MOESM1_ESM.pdf]

Transcriptomic analysis reveals a previously unknown role for CD8^{+} T-cells in rVSV-EBOV mediated protection

Andrea Rivera[1], Suhas Sureshchandra[2], Andrea Marzi[3], Heinz Feldmann[3], Ilhem Messaoudi[4]*

Supplementary Information

Supplementary Figure 1

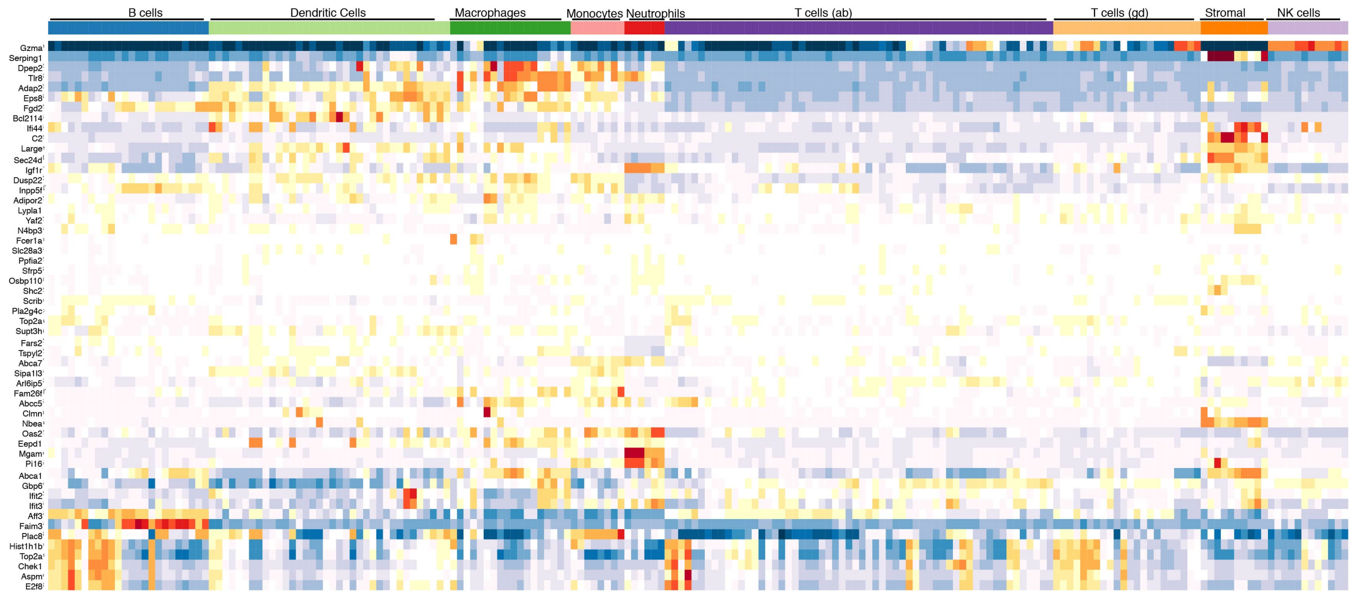

Supplementary Figure 1. Immgen analysis of DEGs 7 days post vaccination. Heatmap profile showing expression profile of genes differentially expressed after rVSV-EBOV vaccination across immune cell populations as predicted by ImmGen's MyGeneSet application. Each row designates a gene and corresponding microarray ID while each column represents a study. Red indicates high likelihood of expression while blue indicates low likelihood of expression within a given immune cell subset.

Supplementary Figure 2

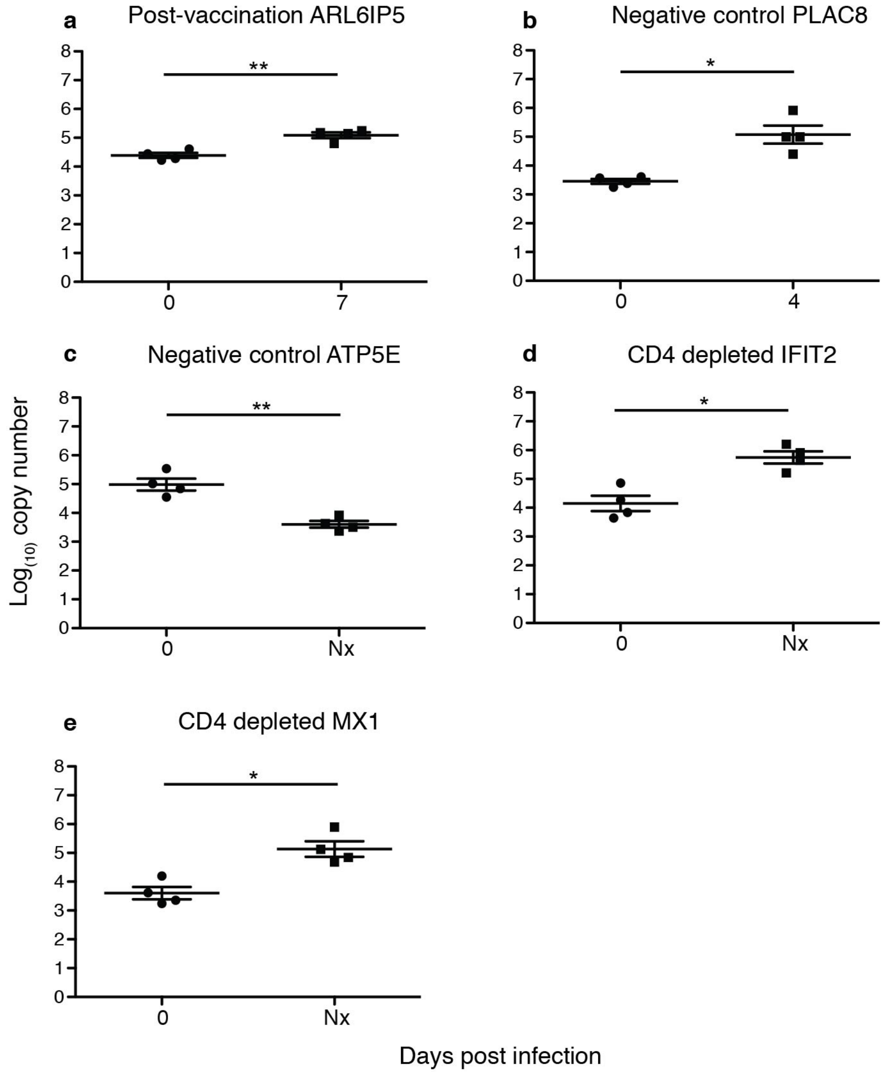

Supplementary Figure 2. qRT-PCR validation of DEGs post-challenge. qRT-PCR expression levels of (**a**) ARL6IP5 in animals 7 days post vaccination with rVSV-EBOV-GP; t-value=8.284; degrees of freedom (df) = 3. (**b**) PLAC8 in negative controls 4 dpi; t-value=4.550; df=3. (**c**) ATP5E in negative controls at necropsy; t-value=6.299; df=3. (**d**) IFIT2 in CD4-depleted animals at necropsy; t-value=3.341; df=3. (**e**) MX1 in CD4-depleted animals at necropsy are shown; t-value=3.641; df=3. Expression of mRNA was normalized to expression of RPL32. Changes in gene expression post infection were

determined using a paired two-sided t-test (n=4 in each group). Means ± are shown. *, $P < 0.05$, **, $P < 0.01$, ***, $P < 0.001$, ****, $P < 0.0001$).

Supplementary Figure 3

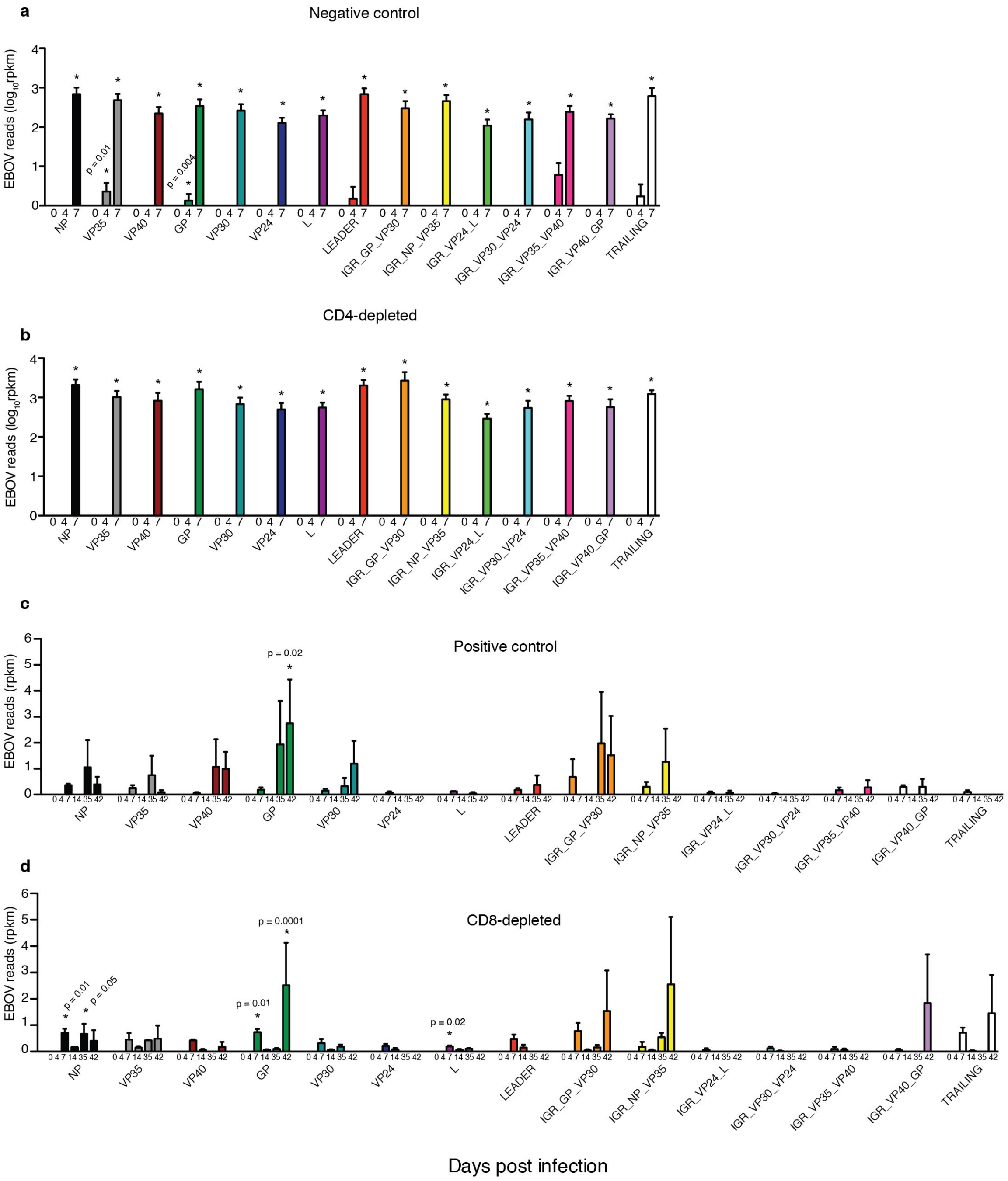

Supplementary Figure 3. EBOV reads mapping to open reading frames and intergenic regions. RPKM normalized RNA-Seq reads mapping to each EBOV open

reading frame (ORF), intergenic region (IGR), as well as leader and trailing sequences at each time point in (**a**) negative controls, (**b**) CD4-depleted animals, (**c**) Positive controls and (**d**) CD8 depleted animals; n=4 in all groups. FDR adjusted p-Value was obtained following *EdgeR* analysis. In both negative control and CD4-depleted animals, (*) denotes a p-Value < 0.0001.

Supplementary Figure 4

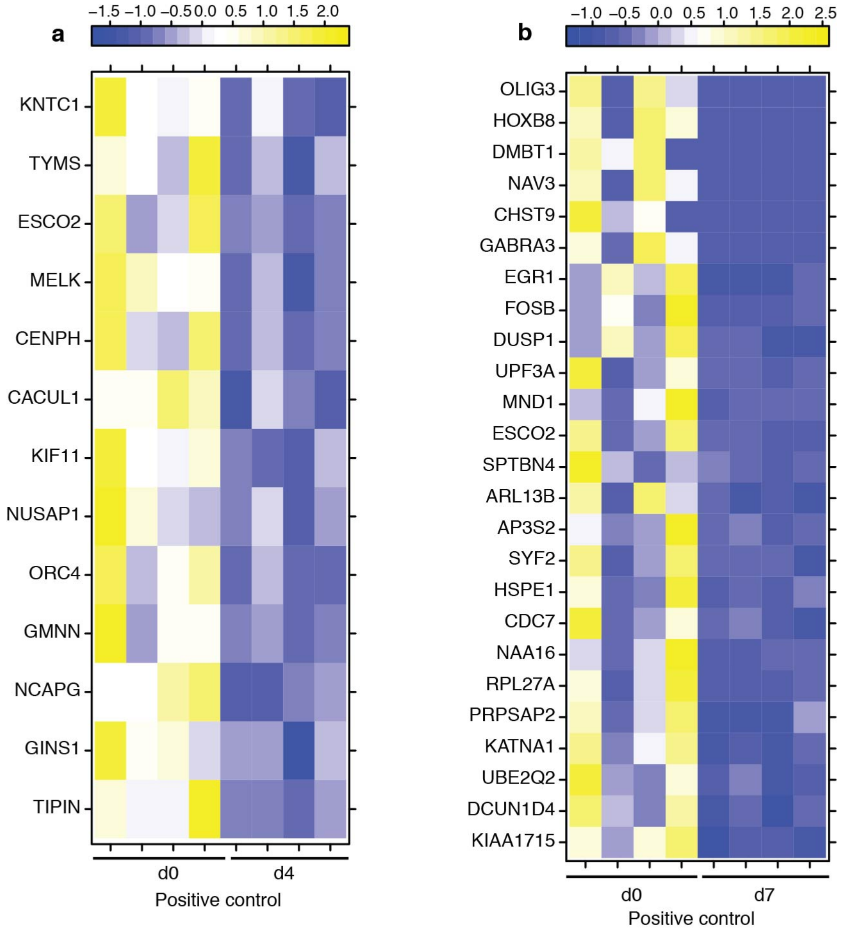

Supplementary Figure 4. Downregulated genes in positive control animals. (**a**) Heatmap of genes downregulated 4 dpi in positive control animals that map to "Mitotic Cell Cycle Process"; each column represents 1 animal in the positive control group. (**b**) Heatmap of all 25 genes downregulated 7 dpi in vaccinated animals.

Supplementary Figure 5

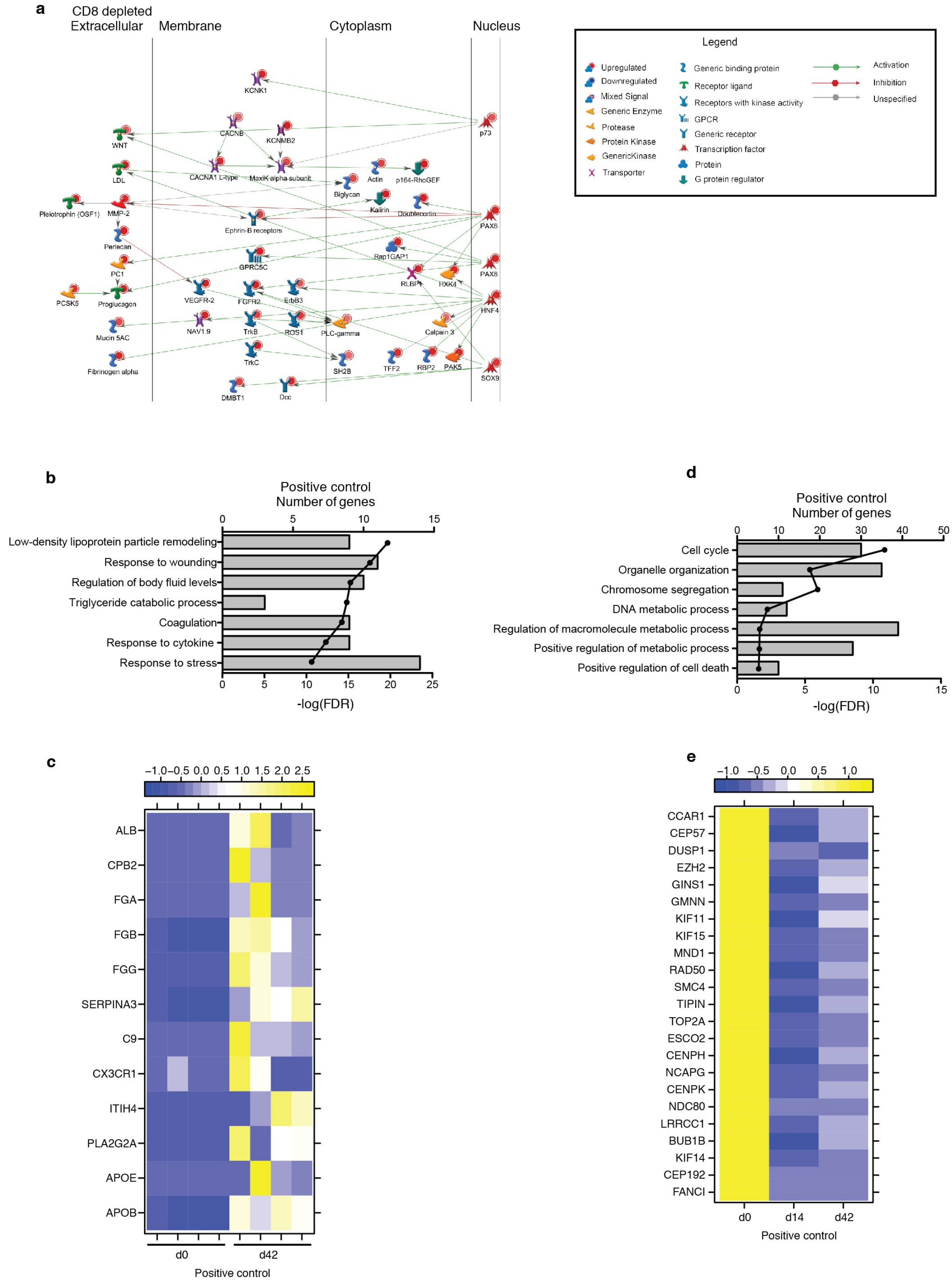

Supplementary Figure 5. DEGs upregulated 35 dpi in CD8-depleted animals and 42 dpi in positive control animals. (**a**) Network depicting direct interactions of genes upregulated 35 dpi in CD8-depleted animals that map to “Signaling”. (**b**) Bar graph depicting the most statistically significant GO terms enriched among up-regulated genes positive control animals 42 dpi (**c**) Heatmap of genes upregulated 42 dpi in positive control animals mapping to “Response to Cytokine” and “Coagulation”; each column represents 1 animal in the positive control group. (**d**) Bar graph depicting the most statistically significant GO terms enriched among down-regulated genes positive control animals 42 dpi. (**e**) Heatmap of down-regulated genes 42 dpi in positive control animals mapping to “Cell Cycle”; each column shows median transcript RPKM counts of all animals in the positive control group at each day.

Supplementary Table 1. Composition of DEGs

**Up-regulated 4 dpi**				
**Category**	**Negative control**	**CD4-depleted**	**Positive control**	**CD8-depleted**
Human homolog	552	115	1	8
ncRNA	97	2	0	0
uncharacterized	74	9	1	9
**Down-regulated 4 dpi**				
**Category**	**Negative control**	**CD4-depleted**	**Positive control**	**CD8-depleted**
Human homolog	950	307	37	2
ncRNA	13	5	63	1
uncharacterized	224	68	12	1
**Up-regulated 7 dpi**				
**Category**	**Negative control**	**CD4-depleted**	**Positive control**	**CD8-depleted**
Human homolog	1013	2074	49	174
ncRNA	36	26	3	32
uncharacterized	444	361	11	137
**Down-regulated 7 dpi**				
**Category**	**Negative control**	**CD4-depleted**	**Positive control**	**CD8-depleted**
Human homolog	814	1174	25	83
ncRNA	29	79	76	6
uncharacterized	246	365	31	52
**Up-regulated 14 dpi**				
**Category**	**Positive control**		**CD8-depleted**	
Human homolog	32		54	
ncRNA	3		15	
uncharacterized	4		8	
**Down-regulated 14 dpi**				
**Category**	**Positive control**		**CD8-depleted**	
Human homolog	295		179	
ncRNA	72		14	
uncharacterized	123		83	
**Up-regulated 35 dpi**				
**Category**	**Positive control**		**CD8-depleted**	
Human homolog	1		502	
ncRNA	0		31	
uncharacterized	0		305	
**Down-regulated 35 dpi**				
**Category**	**Positive control**		**CD8-depleted**	
Human homolog	0		536	
ncRNA	0		7	

uncharacterized	2	101
**Up-regulated 42 dpi**		
**Category**	**Positive control**	**CD8-depleted**
Human homolog	18	117
ncRNA	2	7
uncharacterized	5	27
**Down-regulated 42 dpi**		
**Category**	**Positive control**	**CD8-depleted**
Human homolog	84	352
ncRNA	15	87
uncharacterized	170	136

Supplementary Table 2. Gene ontology enrichment of positive control animals 14 dpi

DPI	Up-regulated	# DEG	FDR adjusted pValue	Down-regulated	# DEG	FDR adjusted pValue
14	Glycogen catabolic process	2	6.959E-02	Cell cycle	77	1.445E-21
	Regulation of metabolic process	19	6.959E-02	Organelle organization	103	2.529E-14
	Biological regulation	25	2.927E+00	Chromosome segregation	24	1.534E-11
	Positive regulation of metabolic process	12	1.010E-01	Cellular macromolecule metabolic process	159	1.753E-11
	System development	13	1.306E-01	Metabolic process	197	3.395E-07
	Regulation of macromolecule metabolic process	15	1.306E-01	Viral transcription	12	2.191E-06

Supplementary Table 3. Gene ontology enrichment of CD8-depleted animals 14 and 35 dpi

DPI	Up-regulated	# DEG	FDR adjusted pValue	Down-regulated	# DEG	FDR adjusted pValue
14	Positive regulation of macrophage derived foam cell differentiation	7	5.404E-09	Cell cycle	37	3.341E-06
	Triglyceride catabolic process	7	1.205E-07	Organelle organization	59	3.341E-06
	Regulation of body fluid levels	14	1.060E-05	Chromosome organization	27	2.678E-05
	Coagulation	12	1.365E-05	DNA metabolic process	23	1.059E-04
	Lymphocyte activation	11	1.365E-05	RNA processing	19	1.866E-03
	Response to virus	8	1.439E-04	Macromolecule metabolic process	92	3.134E-03
	Response to wounding	13	1.447E-04	Cellular protein localization	26	3.228E-03
	Immune system process	21	6.011E-04	Cellular response to stress	33	3.260E-03
35	System development	236	4.277E-23	Organelle organization	213	3.511E-37
	Cell development	127	5.278E-18	Cell cycle	132	1.852E-34
	Regulation of localization	149	5.168E-17	Cellular macromolecule metabolic process	306	2.859E-23
	Cell differentiation	183	4.963E-14	Cell division	62	2.097E-21
	Regulation of ion transport	61	1.063E-13	Chromosome organization	84	1.183E-18
	Single-organism process	437	1.345E-13	Cellular metabolic process	332	3.671E-14
	Circulatory system development	71	9.951E-12	Protein metabolic process	201	8.241E-13
	Signaling	231	4.958E-10	Cellular response to stress	107	5.467E-12
	Chemotaxis	59	1.851E-09	DNA metabolic process	57	1.988E-09
